# Locomotory control in amphioxus larvae: new insights from neurotransmitter data

**DOI:** 10.1186/s13227-017-0067-9

**Published:** 2017-02-16

**Authors:** Thurston Lacalli, Simona Candiani

**Affiliations:** 1 Biology Department, University of Victoria, Victoria, BC Canada; 2 Laboratory of Developmental Neurobiology, DISTAV, Università di Genova, Genoa, Italy

**Keywords:** Amphioxus, Chordate swimming, Intercellular junctions, *Pikaia*

## Abstract

Amphioxus larvae have a midbrain-level locomotory control center whose overall organization is known from serial TEM reconstructions. How it functions has been a puzzle, owing to uncertainty as to the transmitters used by each class of neurons, but this has recently become clearer. We summarize what is now known, and correct past misconceptions: The large paired neurons at the core of the control center are glutamatergic, and hence excitatory, the commissural neurons are GABAergic, hence probably inhibitory, and both motoneurons and ipsilateral projection neurons are cholinergic, suggesting that the latter, a class of interneurons, may be derived evolutionarily from the former. The data clarify some aspects of how fast and slow swimming are controlled and prevented from interfering with one another, but leave open the source of pacemaker activity, which could reside in the large paired neurons or circuits associated with them. A unusual type of non-synaptic junction links the fast and slow systems, but how these junctions function is open to interpretation, depending chiefly on whether they act to couple adjacent cells independent of cell type, or can have differential effects that vary with cell type. Some evolutionary implications are discussed.

## Background

Amphioxus is currently accepted as the closest available model for what basal chordates may have been like, having replaced tunicates in that role [1]. It is a powerful swimmer for its size, relying for that purpose on a similar complement of slow-twitch and fast-twitch muscle fibers to that of vertebrates [2, 3]. Some degree of similarity is thus to be expected between the locomotory control system in amphioxus and that of vertebrates, as the anterior CNS is organized along similar lines [4–6]. In a best-case scenario, understanding how swimming is controlled in amphioxus could provide useful insights into the evolutionary sequence by which a vertebrate-type locomotory control system was assembled. By way of example, the Cambrian fossil *Pikaia* has been interpreted [7] as providing evidence that fast, escape responses were probably a late addition to the behavioral repertoire of basal chordates already capable of a slower mode of undulatory swimming. Amphioxus offers an opportunity to see if the morphology reflects this in any way.

When it comes to neuroanatomical details, the early larva of amphioxus is especially suitable subject for study, being far smaller and simpler in organization than the juvenile and adult. One of us (TL) has carried out a serial TEM analysis of the anterior nerve cord in a 12-day larva of *Branchiostoma floridae* to the end of somite 2 (see [4] for a summary). This is but a small fraction of the nerve cord, but includes the amphioxus counterparts of vertebrate forebrain and midbrain, regions of particular interest. Without complimentary neurotransmitter data, however, interpreting the morphology in functional terms is difficult if not impossible. To date, the most complete analysis of neurotransmitters in the anterior nerve cord is that of Candiani et al. [8], using riboprobes for neurotransmitter marker genes (biosynthetic enzymes and/or vesicular transporters, see [9]) on *B. floridae* larvae up to 3 days in age. Re-examining both TEM and in situ expression data in light of recent observations by Elisabeth Zieger on *B. lanceolatum*, we are now sufficiently confident about cell identity to revisit previously published conclusions and correct some misconceptions. The results provide insight into how the slow and fast swimming modes are controlled, and how interference between them may be prevented. Questions remain, principally about the role played in these processes by an unusual class of juxta-reticular (JR) junctions, which link the two systems, and how both the alternating phase of muscle contraction and its frequency are controlled.

## From TEM: the locomotory control center

**Fig. 1:**
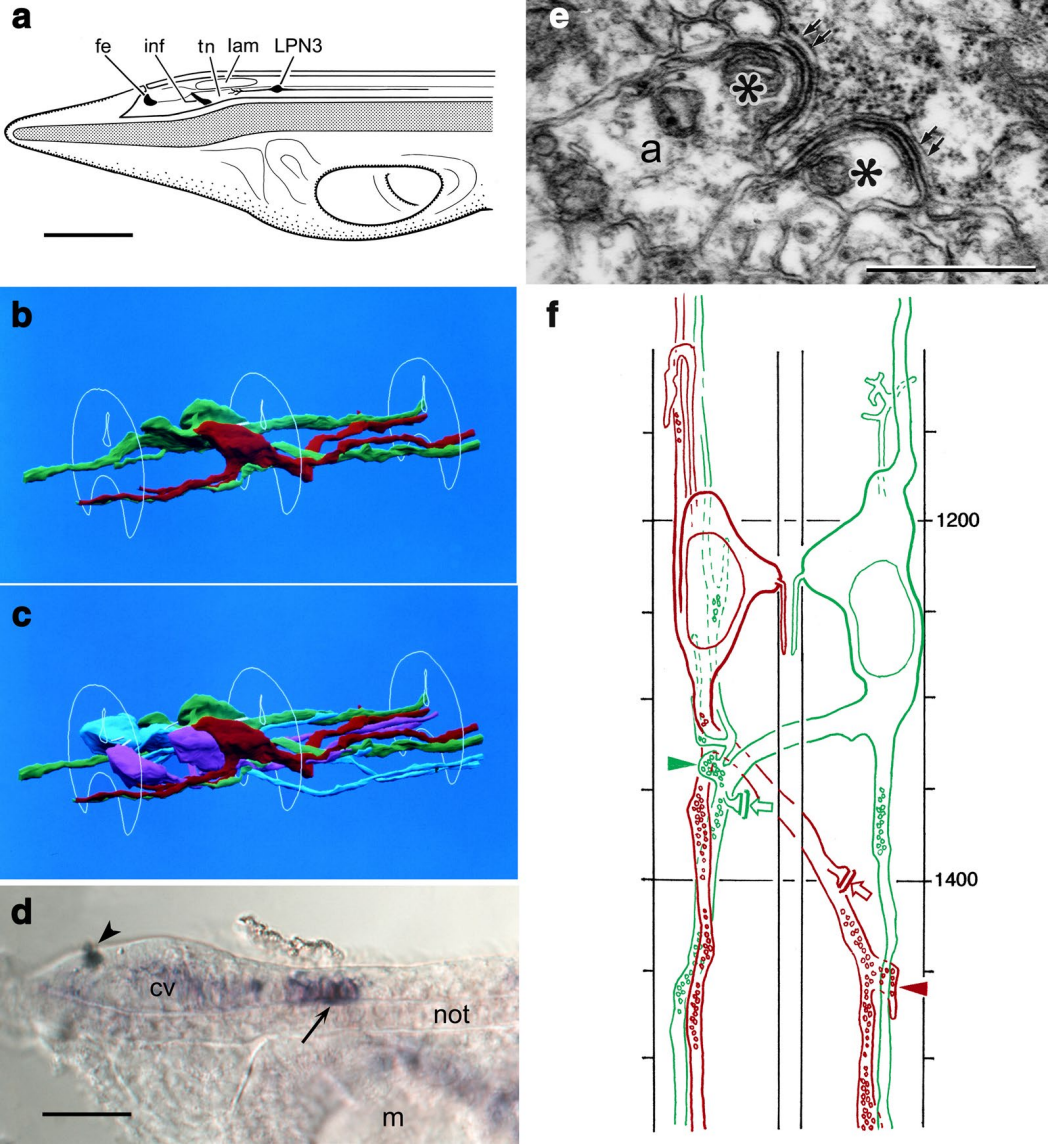
Location and morphology of the third pair of large paired neurons (*LPN3s*). **a** Survey view of the head of a ca. 12-day *B. floridae* larva. The LPN3s are the largest of a group of interneurons that together form the primary motor center. Other anterior landmarks: frontal eye (*fe*), infundibular cells (*inf*), lamellar body (*lam*) and tegmental neuropile (*tn*). *Scale bar* 50 μm. **b** 3D reconstruction of the two LPN3s (*red* and *green*) from serial TEM of a 12-day larva, showing the nerve cord in outline, anterior to the left, with dendrites projecting forward and axons behind. **c** 3D reconstruction, as above, with the LPN1s and 2s added (*magenta* and *light blue*), dendrites not shown. **d** Vesicular glutamate transporter (VGLUT) mRNA expression in a 4-day larva, a previously unpublished image using the same riboprobe as in [8], showing increased cell numbers in the LPN cluster (*arrow*). This indicates that LPN1s, 2s and 3s share the same transmitter, i.e., that all three pairs are glutamatergic. Other features: frontal eye pigment spot (*arrowhead*), cerebral vesicle (*cv*), mouth (*m*) and notochord (*not*). *Scale bar* 20 μm. **e** TEM image of a JR junction, in this case a double junction (*asterisks*) between a DC motor axon (*a*) and an IPN cell body, upper right. *Double arrows* mark ER cisternae, with matching cisternae on the *asterisk* side; see [10] for further examples and details. *Scale bar* 0.5 μm. **f** Dorsal view of the two LPN3s in (**b**) in matching *colors*; section *numbers* for the series are on the *right*, marked at 50-section intervals. *Arrowheads* indicate the reciprocal synapses between cells, and *small arrows* the JR junctions they form with the adjacent cell bodies of the first pair of DC motoneurons; see [10, 13] for details. *Scale* 50 sections = 3.4 μm

TEM analysis has revealed one especially prominent group of ventral neurons in the anterior nerve cord, the large paired neurons (LPNs), located in the primary motor center (PMC) at the level of the junction between somites 1 and 2. The third pair of LPNs (LPN3s, Fig. 1) is conspicuous for the large size of their cell bodies and axons, and is distinctive morphologically in other respects: each has both a contralateral and an ipsilateral axon-like projection, the former being both larger and synaptically more active, with an especially large synapse to the ipsilateral projection of the opposite member of the pair where these projections first meet (Fig. 1f). Both also form non-synaptic juxta-reticular (JR) junctions with the cell bodies of the first of the dorsal compartment (DC) motoneurons, which are positioned so as to link each DC cell body with the most proximal part of the corresponding contralateral LPN3 axon. The defining feature of these junctions is the close apposition of a layer of endoplasmic reticulum along the cytoplasmic face of both membranes ([10], see Fig. 1e). Besides the connection between the LPN3s and DC motoneurons, junctions of this type have so far only been found between the DC motoneurons and a class of interneurons known as ipsilateral projection neurons (IPNs), and between successive members of the DC motoneuron series on each side. ER cisternae occur in other taxa at some synaptic sites (e.g., in insect CNS [11]), but the symmetrical arrangement seen in amphioxus has not been reported elsewhere to our knowledge.

DC motoneurons innervate the slow-twitch muscle fibers responsible for the swimming mode used for migration, which for larvae means vertical migration in the water column. Six pairs of DC motoneurons are distributed in a periodic pattern extending from the front of somite 2 to somite 6 [12]. The larval escape response depends on more rapid undulations of greater amplitude, generated by fast-twitch muscle fibers innervated by ventral compartment (VC) motoneurons. These are distributed along the length of the cord, and typically innervate several somites each in an overlapping fashion [3]. Projections from all the somatic motoneurons, DC and VC, are ipsilateral.

The centrality of the LPN3s to control over the escape response is implied by the massive sensory input these cells receive (see Fig. 5 in [13]), by the size of their cell bodies, axons and terminals compared with other anterior neurons, and by the core position their dendrites occupy in the ascending tracts, an indication that they are among the first neurons of the locomotory complex to establish dendritic connections. The temptation is to assume that they are central controllers of all aspects of escape behavior, but this does not necessarily follow. Activating the response is only the initial step, after which both the frequency and phasing of neural output need to be controlled, the latter so that muscles on opposite sides of the body contract in an alternating sequence. For convenience in this account, frequency and phasing are combined and treated together simply as a pacemaker function. This could be circuitry-based and local to the PMC or involve cells elsewhere in the anterior nerve cord, or it could depend on a pattern of rhythmic firing intrinsic to the LPNs themselves. Pacemaker circuits often incorporate cross-inhibition between a matched pair of neurons, which prompted Lacalli and Kelly [13] to suggest that the reciprocal synapses between the LPN3s, if inhibitory, would allow them to perform this function. The first indication this was unlikely was the report by Candiani et al. [8] of a pair of glutamate-containing neurons at the somite 1/2 junction, precisely where the LPN3s are located, along with evidence that nearby neurons were cholinergic. These observations (see Comment 1 to [8]) make it unlikely that either the LPN3s or any of the neurons most closely associated with them are capable of generating a direct inhibitory effect by a conventional synaptic mechanism. The nature of the pacemaker has since remained a puzzle, until recently, when additional data on the expression of markers for each of the main transmitters have allowed us to map the latter onto the TEM data with greater confidence.

## Matching TEM with neurotransmitter data

**Fig. 2:**
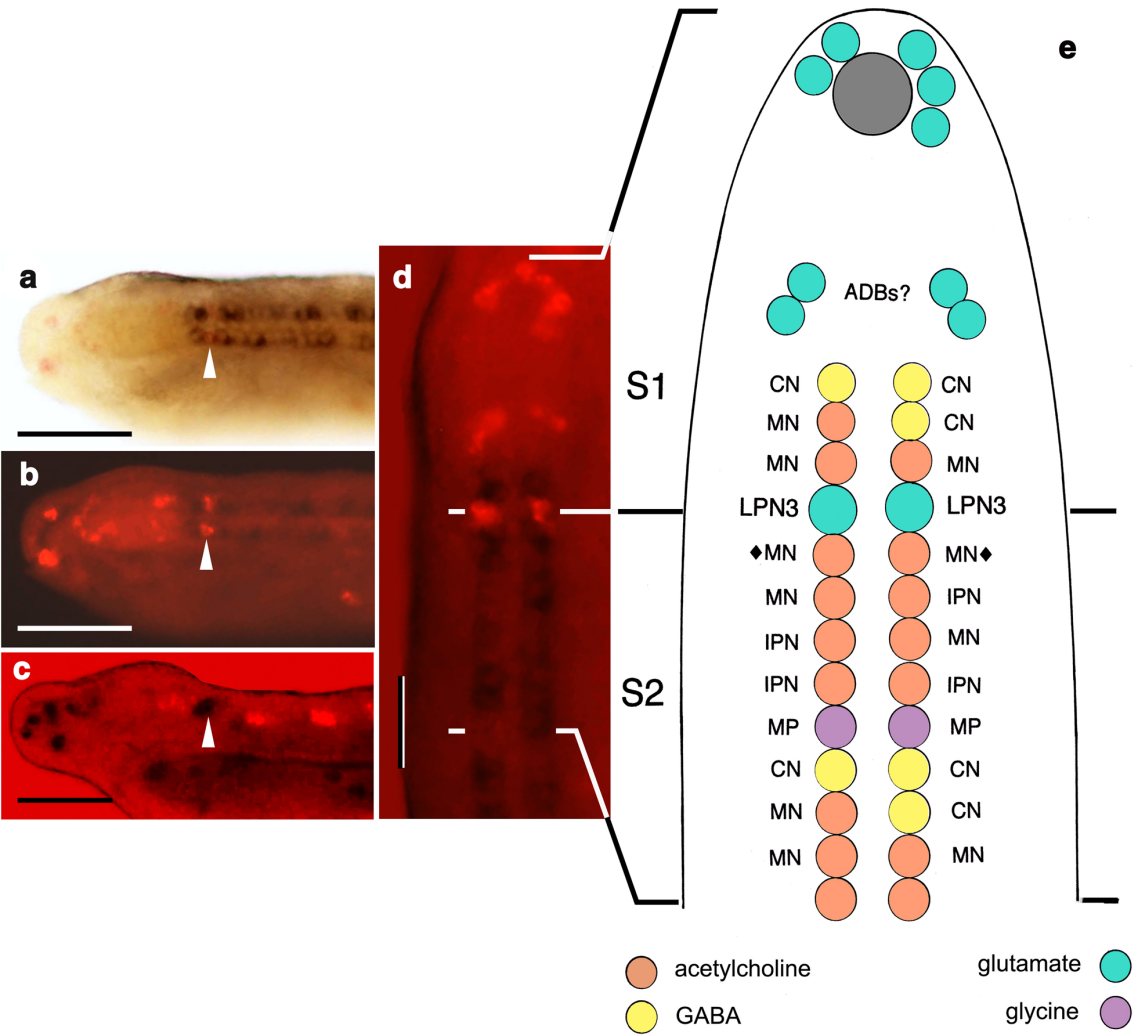
In situ data on transmitter localization. **a**, **b** Images from Fig. 10g–i in [8], showing VAChT (*dark purple*) and VGLUT (*red*) expression, markers for cholinergic and glutamatergic neurons, respectively. **c** Image from Fig. 9 g in [8], showing VGLUT expression in *dark purple* and VGAT (GAD+) expression in *red*, markers for glutamatergic and GABAergic neurons, respectively. *Arrowheads* in (**a**–**c**) indicate the position of the LPN3s; the loose cluster of red cells just forward of this point in (**c**) are the putative anterior group CNs. *Scale bars* for (**a**–**c**) 50 μm. **d** A dorsal view of the specimen in (**a**) reoriented for comparison with the diagram; the junction between somites 1 and 2 corresponds with the position of the LPN3s as shown. *Scale bar* 5 μm. **e** Revised neurotransmitter map, an update combining Figs. 3 and 12 from [8] showing the anterior end of the dorsal nerve cord in 20–24 h neurulae of *B. floridae* to the end of somite 2, opened out and viewed from above, with each of the ventral neurons expressing markers for known transmitters matched with the corresponding cell type from TEM. Neurons: commissural neurons (*CN*), third pair of large paired neurons (*LPN3*); motoneurons *(MN*, *diamonds* mark the first pair of DC motoneurons), multipolar neurons (*MP*), ipsilateral projection neurons (*IPN*). The *color code*, by transmitter, is shown at the *bottom*. The figure is altered from the original to show how the anterior-most glutamatergic neurons are positioned at 24 h based on (**d**). Several such cells cluster around the anterior pigment spot (*gray*) belonging to the frontal eye, but we are uncertain as to their identity. We interpret the paired dorsolateral groups of cells between the pigment spot and the ventral columns as anterior dorsal bipolar cells (*ADBs*) as indicated

There are two problems comparing TEM and neurotransmitter data: (1) that the stages differ, so there may be neurons identified by TEM at 12 days that will not as yet have differentiated at the stages used for in situ preparations and (2) that, based on a single TEM specimen, we have no measure of variability between specimens for the cells identified by that means alone. To date, the most reliable landmark has proven to be the pair of glutamatergic neurons located at the junction between somites 1 and 2 (Fig. 2a–d), the pair of LPN3s identified by TEM being its only plausible counterpart. A question then arises about the LPN1s and 2s, which lie just forward of the LPN3s (Fig. 1c). Comparing in situ data from 3- and 4-day specimens shows that these cells first appear at 4 days (Fig. 1d), and are also glutamatergic, but the timing of their development means they would not be expected in cell maps based on 2- and 3-day specimens. This simplifies the in situ analysis, in that we are then left with 3–4 GABA-containing neurons and a similar number of cholinergic neurons immediately forward of the LPN cluster, the exact order being somewhat variable between specimens, plus a column of 3–4 cholinergic neurons on each side caudal to the LPN3s followed, about midway along somite 2, by three GABA-containing neurons, two on the right and one on the left. Comparing with TEM data, the motoneurons immediately anterior to the LPNs account for the cholinergic neurons in that region, so by default, the anterior group of GABA-containing neurons are most likely the commissural neurons (CNs) located forward of the LPNs. For the region caudal to the LPNs, the GABA-containing neurons could be CNs or IPNs, but choosing between these is difficult given limited knowledge regarding how variable either is in number and position between specimens.

A key observation made recently by E. Zieger (pers. communication) on *B. lanceolatum* is that the GABAergic neurons, which are very similarly positioned from specimen to specimen in both species, have axons that invariably cross the midline. This confirms their identity as CNs and excludes the IPNs, which have only ipsilateral projections. Lacalli and Kelly [13] report four putative CNs in somite 1 (see their Fig. 9a), and further tracing has revealed three similar cells near the midpoint of somite 2, two of which are on the right, matching the GABA data. While there are other neurons in the TEM specimen with crossing fibers, all those identified as CNs have a similar complement of vesicles (see [13]), close contact with other CN axons where they cross (Fig. 10a in [13]), and dendrites that track the LPN3 axons, forward in the case of somite 2 CNs, and as branches from caudally directed axons in the case of somite 1 CNs. From this and other features of the axons, including their location within the neuropile, we are quite confident that the GABAergic neurons correspond with the CNs identified by TEM.

This leaves just enough cholinergic neurons on each side in *B. floridae* in situ preparations to account for the VC and DC motoneurons and the IPNs. There is then a single pair of glycinergic neurons in somite 2, positioned between the last of the anterior file of cholinergic neurons and the first of the GABAergic neurons, for which the best match is the pair of multipolar neurons (MPs) described by Lacalli and Kelly (Fig. 9d in [13]). These are unusual in having axons that branch, cross to both sides of the cord and travel both forward and back. Their synaptic targets include motoneurons and an assortment of interneurons that, assuming the MPs are glycinergic and inhibitory, implies a role in inhibiting the locomotory system at multiple points.

On the above evidence, we can now assign all the ventral neurons reported by Candiani et al. [8] in somites 1 and 2 as shown in Fig. 2e. Both GABAergic and glycinergic neurons prove to be distinctive morphologically, but surprisingly the IPNs, a class of interneuron, are cholinergic. We interpret this as evidence that the IPNs may be secondarily derived from motoneurons, as they have a remarkably similar morphology, including ipsilateral axons that track at similar levels in the neuropile, and short dendritic spines distributed all along the axon. The latter feature is entirely atypical of other ventral interneurons, at least at this early stage of development.

## Interpreting the circuitry

**Fig. 3:**
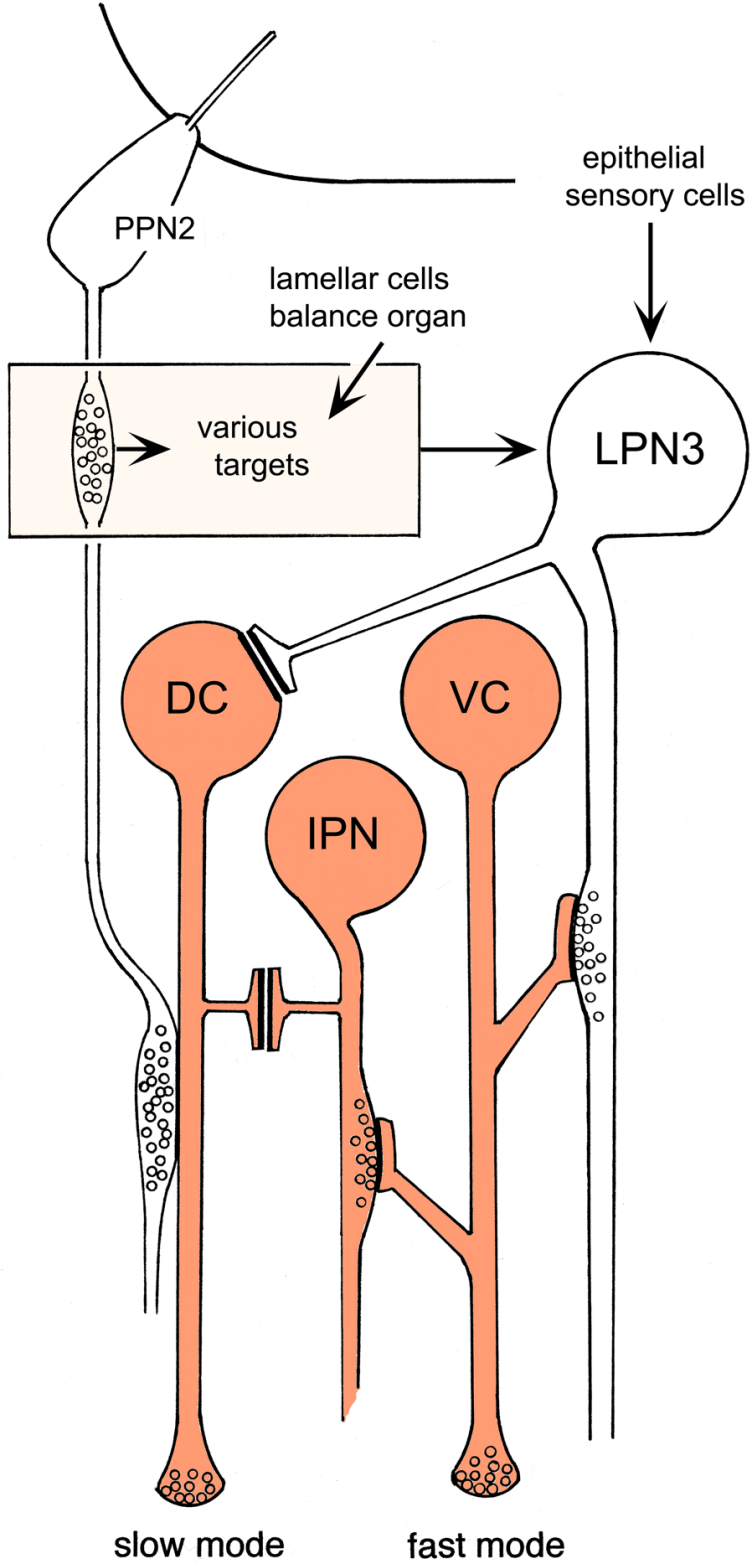
Inputs to the DC and VC motoneurons. A schematic summary diagram, data from somites 1 and 2 in a 12-day *B. floridae* larva, showing synaptic inputs (*arrows* and *terminals with vesicles*) and contacts via JR junctions (*parallel lines*) to the dorsal compartment (*DC*) and ventral compartment (*VC*) motoneurons. Other neurons: third pair of large paired neurons (*LPN3*), ipsilateral projection neurons (*IPN*), type 2 preinfundibular projection neurons (*PPN2*). The tegmental neuropile (*tn*) is a region where paracrine transmission predominates. Neurons colored orange are cholinergic, as in Fig. 2e. Modified from [10]

Knowing the transmitters, we now have a much clearer idea of how the locomotory control circuits operate. Assuming that acetylcholine and glutamate are excitatory transmitters in amphioxus, as in other chordates, the input to the VC motoneurons responsible for the escape response from LPNs and IPNs is excitatory. Inhibitory inputs come from the CNs and MPs, which we suspect, based on projection patterns, are concerned primarily with controlling the phase and/or strength of contractions (in the case of CNs) or suppressing swimming altogether (MPs). Here, we are more concerned with the activation circuits (Fig. 3), which show a clear separation between the fast (VC) and slow (DC) systems. Excitatory synaptic inputs to the former are from epithelial sensory cells, whose fibers enter the cord via the rostral and dorsal nerves, from anterior dorsal bipolar cells (ADBs), and from LPNs and IPNs. The DC system, in contrast, receives no synaptic input from these cells so far as we know, but is instead linked to the LPNs and IPNs by JR junctions positioned (Fig. 1f) so contact with the former occurs where the contralateral projection from each LPN3 meets the anterior-most DC motoneuron on the opposite side.

JR junctions also link the first and second of the DC motoneurons on each side [10], so we assume that the entire DC series may be similarly linked, either one cell to the next or the first cell to the entire series. This arrangement is consistent with the JR junctions having a role in coupling cells functionally so they act together, and implies the junctions locally depolarize the cell or axon to which they connect to excite them. There would be a problem, however, if the junctions to the LPN3s and IPNs behaved in this fashion, since activating the fast system would then also activate the slow system and vice versa. A way to avoid this would be if the junctions operate differently when only one of the two linked cells is a DC motoneuron, rather than both. Assuming this may be the case, two further options need to be considered.

The first is the possibility that the potential generated at the junction between a DC motoneuron and either an LPN or IPN results in mutual inhibition. Activation of the LPN3s by sensory input would then activate the VC motoneurons by synaptic means, while simultaneously inhibiting the first of the DC neurons on each side via junctions. Activating the DC system via its normal inputs, e.g., from the PPN2s, and possibly other sources we have not yet identified, would block transmission in the proximal part of each of the contralateral LPN3 axons. It could also, by the same mechanism, block the firing of the excitatory IPNs, thus suppressing the VC system by two routes. In short, each system, once activated, would suppress the other. Separating the pathways in this way would necessarily mean there are two separate pacemakers, one to drive the fast mode and a second for the slow mode. The former could be circuitry-based, as there is an assortment of early-developing neurons with ascending fibers in somites 4 and 5 that could be involved in coordinating this function [13], but it is also possible that the LPN3s have an intrinsic capability to fire in a rhythmic fashion. As to the DC pacemaker, if it is truly independent of the VC system, the morphology so far provides no clues as to where it might be located or how it operates. Further, it is not clear how DC motoneurons on opposite sides of the cord would coordinate their activity, as all other inputs so far identified to them are from ipsilateral projections of cells that themselves lack contralateral inputs. A variant on the inhibitory option would be to have the junctional block operate in one direction only, e.g., between the DC motoneurons and LPN3 but not the reverse. It is not, however, clear to us how this would produce a functionally useful result.

A second option is to assume the DC motoneurons are connected to the LPN3s by junctions to allow the LPN3s act as pacemakers for slow and fast swimming. To accomplish this, the junctional potential produced when the DC motoneurons are active would have to cause a reduction in pacemaker frequency, from fast to slow. In addition, some means would be needed to prevent the VC system from operating at this slower rate. The latter could be achieved simply through a threshold effect: Escape behavior is evoked in normal circumstances by highly redundant sensory inputs to multiple excitatory pathways, not just the LPNs, so synaptic inputs from the latter may not be sufficient on their own to trigger an escape response. If the DC neurons are able also to suppress the IPNs via junctions, a further source of excitatory input to the VC system is removed, which could be enough to block the fast response entirely. In fact, it is possible that the IPNs evolved for this very reason, to provide a sufficient proportion of the excitatory synaptic input to the VC motoneurons, that its loss prevents their being activated by other pathways. The other problem is to prevent the DC system from being activated when the LPN3s are operating in fast mode, but this again could be a threshold effect, with the DC motoneurons being unresponsive when the other inputs to them fall below some threshold.

How likely either of the above options is, in physiological terms, is not immediately obvious to us. Nor is it easy to assess them by comparison with the neural mechanisms that underlie undulatory swimming in vertebrates. There are similarities, e.g., excitatory descending interneurons play a central role in locomotory control in *Xenopus* larvae [14] and, like the LPNs, are glutamatergic, but they differ from the latter both in morphology (their projections are ipsilateral rather than contralateral) and in location (hindbrain rather than midbrain). Electrical coupling between cells also plays a role in locomotory control in *Xenopus* larvae and other vertebrate systems [15], but this involves gap junctions rather than the specialized junctions we find in amphioxus. Lacking comparable electrophysiological data for the amphioxus system, we are at a considerable disadvantage when it comes to assessing how closely locomotory control circuits in amphioxus resemble those in vertebrates. It is also not clear whether there is any relation between the LPNs and any of the giant brainstem neurons identified in vertebrates, e.g., Mauthner neurons [16] or the Müller cells of lampreys [17]. LPNs are also considerably smaller than the giant Rohde cells that coordinate some aspects of swimming in adult amphioxus, consistent with the idea that giant cell systems have evolved multiple times among chordates. Caution is therefore required in assessing the degree of homology between them.

Despite a limited knowledge of the physiology, what we do know of locomotory control in amphioxus larvae has evolutionary implications that are worth considering. Based on an interpretation of the Cambrian fossil *Pikaia*, a case can be made for basal chordates being slow swimmers with limited abilities for fast escape from predators [7]. If the organization of the DC (slow) system in amphioxus is any guide, early chordates would have had a dedicated group of neurons in the anterior segments responsible for initiating slow waves of contraction that propagate along the rest of the trunk and tail. Stimulating more localized contractions, a potentially useful way to produce greater force at particular points, would have required a separate set of motoneurons that could be independently controlled. The VC (fast) system could have begun this way, providing a means to locally adjust contraction strength during slow mode swimming, eventually to evolve into a separate set of motoneurons dedicated specifically to fast escape. For the first option discussed above, where junctions isolate the two systems and provide a mutual block, one can envisage the PMC neurons evolving in parallel with the VC motoneurons as the latter became more important, and the junctional block evolving simultaneously so the two swimming modes would not interfere. This would imply that the PMC is a comparatively late innovation, at least as regards its central role in initiating, and perhaps coordinating the fast response. The second option, where either the LPN3s themselves or circuits in which they participate serve as pacemakers for both systems, implies that the PMC is considerably older. It would have to be at least as old as somites and undulatory locomotion, unless the reliance of the slow system on an LPN3-based pacemaker evolved secondarily to replace a separate and earlier slow pacemaker that has since been lost.

There is no way currently to choose between the above options, and our intention in discussing them is less to argue for one over the other, than to point out the difficulty of judging either until we understand more clearly how the pacemaker or pacemakers, if there are two, are organized and function.

## Conclusions

The neurotransmitter data on the early larval nerve cord in amphioxus is now sufficiently complete that we can assign transmitters to the principal ventral neurons identified by TEM with some confidence. The pathways that initiate the escape response can then be better understood: Synaptic input from glutamatergic neurons is especially important, including from epithelial sensory cells and the large paired neurons (LPNs) that are core components of the locomotory control center. In contrast, the motoneurons responsible for slow swimming are isolated from these excitatory pathways and, so far as we know, are linked to the escape pathway only by non-synaptic JR junctions located at strategic points. How the two systems coordinate, so that interference is avoided, is currently not clear. In assessing the alternatives, a crucial point is the nature of the junctions themselves, their mechanism of action, and whether the fast and slow mode each has its own pacemaker. They could instead share a single pacemaker, possibly acting through the LPNs, but only if the frequency can be modulated by a non-synaptic mechanism, possibly involving JR junctions.

## Abbreviations

ADB: anterior dorsal bipolar neuron
CN: commissural neuron
DC: dorsal compartment (motoneuron)
ESC: epithelial sensory cell
fe: frontal eye
GABA: γ-aminobutyric acid
GAD: glutamic acid decarboxylase
inf: infundibular cells
IPN: ipsilateral projections neuron
JR: juxta-reticular (junction)
lam: lamellar body
LPN1–3: large paired neuron (pairs 1–3)
MN: motoneuron
MP: multipolar neuron
PMC: primary motor center
PPN2: type 2 preinfundibular projection neuron
TEM: transmission electron microscopy
tn: tegmental neuropile
VAChT: vesicular acetylcholine transporter
VC: ventral compartment (motoneuron)
VGAT: vesicular GABA/glycine transporter
VGLUT: vesicular glutamate transporter
